# Supplementary material for: Cyto-Genotoxic and Transcriptomic Alterations in Human Liver Cells by Tris (2-Ethylhexyl) Phosphate (TEHP): A Putative Hepatocarcinogen
Source: Int J Mol Sci. 2022 Apr 3;23(7):3998. doi: 10.3390/ijms23073998 (PMC8999606; doi:10.3390/ijms23073998)
Supplement: Supplementary file 1 [file ijms-23-03998-s001.zip › Supplementary Figure S1.pdf]

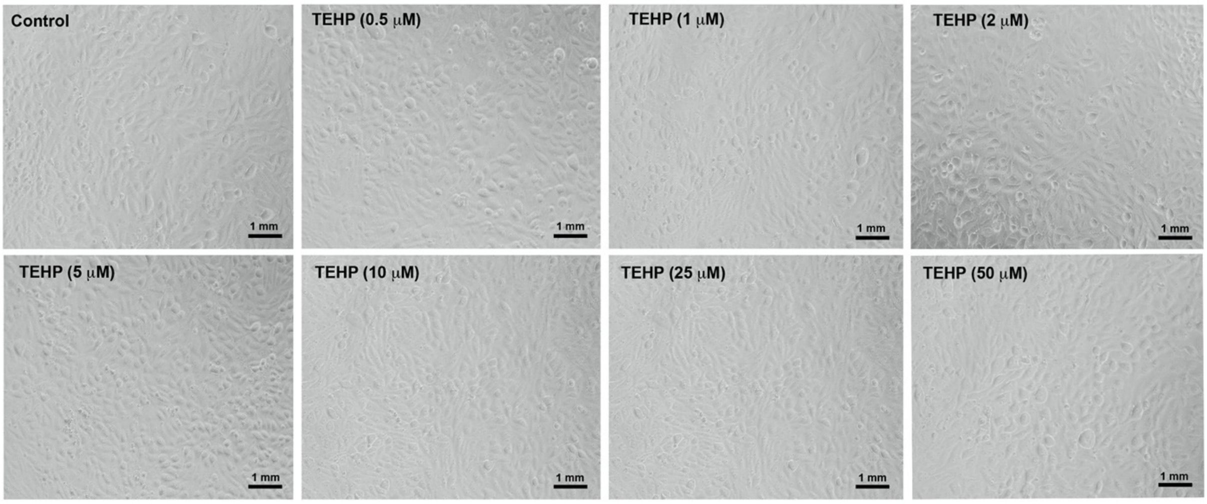

Supplementary Figure S1. Structural changes in HepG2 cells after 72h of exposure with at low concentrations of TEHP.

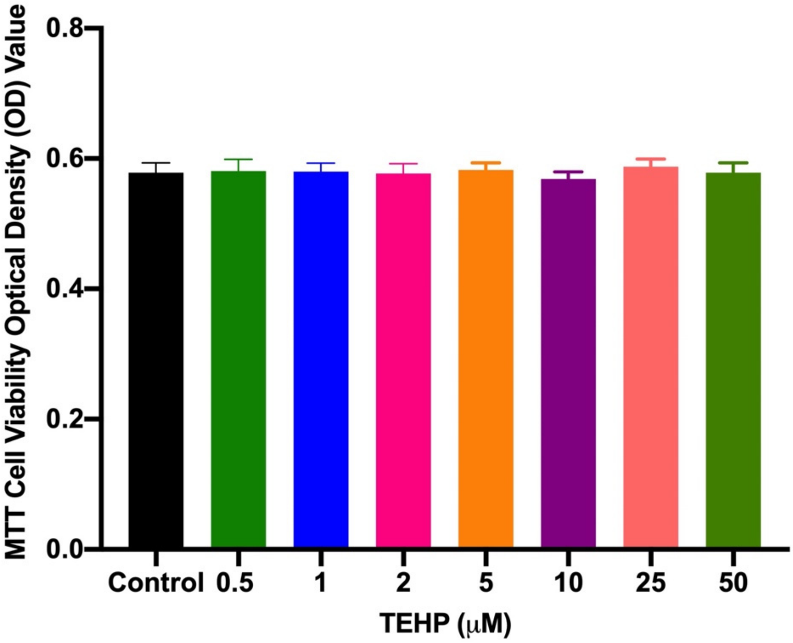

Supplementary Figure S2. Mitochondrial dehydrogenase-based cytotoxicity quantification in HepG2 cells after TEHP exposure for 72 h.

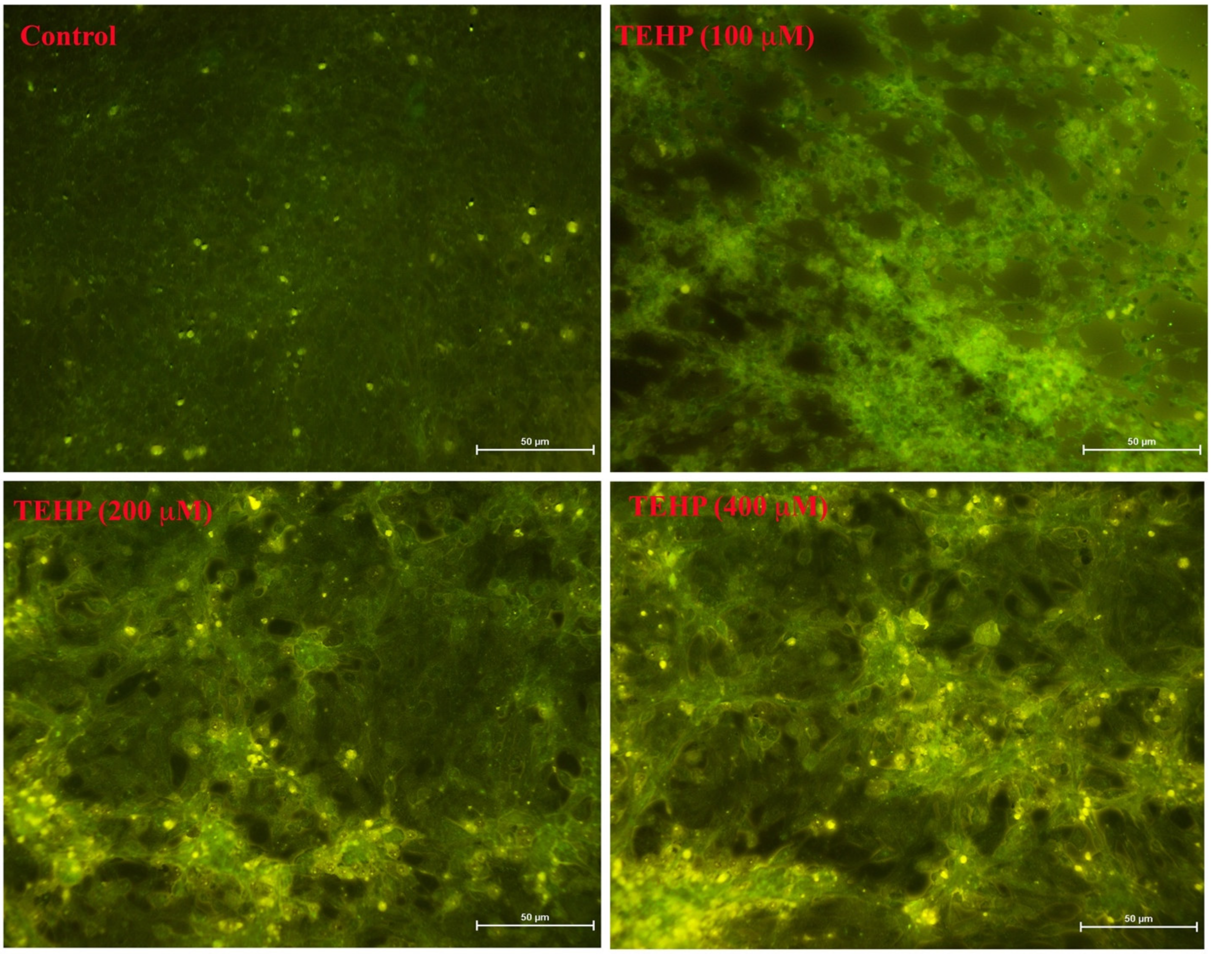

Supplementary Figure S3. Ca^{++} influx in HepG2 cells after TEHP (100-400 μM) treatment for 72h. Images were captured using a fluorescence microscope at 20X. Images depict an increase in the green fluorescence in TEHP treated cells stained with Fluo-3 (0.5 μM) for 1h. HepG2 cells were grown on 16-wells sterile cell-culture glass slide in a CO_2 incubator (5%, 95% humidity, 37 °C).
